# Obstetric fistula repair failure and its associated factors among women who underwent repair in sub-Saharan Africa. A systematic review and meta-analysis

**DOI:** 10.1371/journal.pone.0295000

**Published:** 2024-02-05

**Authors:** Habtamu Endashaw Hareru, Zemachu Ashuro, Berhanu Gidisa Debela, Mesfin Abebe

**Affiliations:** 1 School of Public Health, College of Medicine and Health Sciences, Dilla University, Dilla, Ethiopia; 2 Department of Environmental Health, College of Medicine and Health Sciences, Dilla University, Dilla, Ethiopia; 3 Department of Midwifery, College of Medicine and Health Sciences, Dilla University, Dilla, Ethiopia; University of the Witwatersrand, SOUTH AFRICA

## Abstract

**Background:**

Obstetric fistula repair failure can result in increased depression, social isolation, financial burden for the woman, and fistula care programs. However, there is limited, comprehensive evidence on obstetric fistula repair failure in Sub-Saharan African countries. This systematic review and meta-analysis aimed to determine the pooled prevalence of obstetric fistula repair failure and associated factors among women who underwent surgical repair in Sub-Saharan African countries.

**Methods:**

To identify potential articles, a systematic search was done utilizing online databases (PubMed, Hinari, and Google Scholar). The Preferred Reporting Items for Systematic Review and Meta-Analysis Statement (PRISMA) guideline was used to report the review’s findings. I^2^ test statistics were employed to examine study heterogeneity. A random-effects model was used to assess the pooled prevalence of obstetric fistula repair failure, and the association was determined using the log odds ratio. Publication bias was investigated using the funnel plot and Egger’s statistical test at the 5% level of significance. Meta-regression and subgroup analysis were done to identify potential sources of heterogeneity. The data were analyzed using STATA version 17 statistical software.

**Results:**

A total of 24 articles with 9866 study participants from 13 Sub-Saharan African countries were included in this meta-analysis. The pooled prevalence of obstetric fistula repair failure in sub-Saharan Africa was 24.92% [95% CI: 20.34–29.50%]. The sub-group analysis by country revealed that the highest prevalence was in Angola (58%, 95% CI: 53.20–62.80%) and the lowest in Rwanda (13.9, 95% CI: 9.79–18.01%). Total urethral damage [OR  =  3.50, 95% CI: 2.09, 4.91], large fistula [OR = 3.09, 95% CI: (2.00, 4.10)], duration of labor [OR = 0.45, 95% CI: 0.27, 0.76], and previous fistula repair [OR = 2.70, 95% CI: 1.94, 3.45] were factors associated with obstetric fistula repair failure.

**Conclusion:**

Women who received surgical treatment for obstetric fistulas in Sub-Saharan African countries experienced more repair failures than the WHO standards. Obstetric fistula repair failure was affected by urethral damage, fistula size, duration of labor, types of fistula, and history of previous repairs. Therefore, we suggest policy measures specific to each country to provide special attention to the prevention of all risk factors, including poor nutrition, multiparty, obstructed labor, and maternal age, which can result in conditions like large fistulas, urethral damage, and repeat repair, in order to reduce obstetric fistula repair failure.

## Introduction

An obstetric fistula is defined by the World Health Organization (WHO) as "an abnormal opening between a woman’s vagina and bladder and/or rectum through which her urine and/or feces continuously leak" [1]. These openings can be vesico-uterine (between the bladder and uterus), rectovaginal (between the rectum or colon and the vagina), ureterovaginal (between the ureter and the vagina), and/or vesicovaginal (between the anterior vaginal wall and posterior bladder) [2]. Obstetric fistula is frequently the result of obstructed labor, which happens when the baby’s head gets caught in the mother’s pelvis, obstructing blood flow to the neighboring tissues and leading to tissue necrosis, which can result in a fistula [2, 3]. In developing countries, aside from obstructed labor, other factors that contributed to the occurrence of obstetric fistulas included a lack of skilled birth attendants, poor health-seeking behavior, a poor referral system, poor transportation, age and physical maturity, iatrogenic surgical damage, sexual violence, poverty, a lack of awareness, and not spacing out pregnancies [4–6].

The WHO estimates that obstetric fistulas affect between 50 000 and 100 000 women annually. It is predicted that 30 000–130 000 new cases of obstetric fistulas develop each year in Africa alone [7], with over 2 million young women in Asia and Sub-Saharan Africa (SSA) not obtaining treatment for their condition [8]. In nearly 60 countries, it is challenging. Additionally, obstetric fistula can be found in all developing nations, including those in Africa, the Middle East, and Asia. It is most common in the region of SSA known as the fistula belt, which spans the northern half of the continent from Mauritania to Eritrea [9].

Obstetric fistulas have major physical, social, and psychological effects if early, efficient medical care is not received. It causes fecal, urinary, or both types of incontinence [10, 11]. The vulva and thighs may get damaged as a result of ongoing urine and feces leakage [2]. In addition, women with obstetric fistulae are more likely to experience social isolation and marginalization [12, 13], high rates of divorce or separation [14, 15], lack of sexual desire [13, 14], loss of fertility and amenorrhea [16], depression [15, 17], associated comorbidities, reduced self-esteem, and income loss [10, 11, 18].

Obstetric fistula has been a neglected public health and human rights issue despite the fact that it is currently almost entirely preventable and is a sign that social and health systems are failing to protect the health and human rights of the poorest and most vulnerable women and girls. It also persists as a reminder of egregious injustices, a symbol of global inequality, and a sign that these women’s and girls’ rights are not being protected [19, 20].

Obstetric fistula repair surgery is the only proven treatment for the condition, yet results can vary widely based on the patient’s location and the healthcare setting. Repairing an obstetric fistula is essential for a woman’s general health and well-being. The treatment of an obstetric fistula necessitates a precise diagnosis, preoperative care, quick repair, application of fundamental surgical concepts, postoperative care, and follow-up. In addition to causing pain, both physical and psychological, failing to repair a fistula costs money and jeopardizes efforts to end this treatable and preventable illness. The United Nations (UN) has also established a goal to eliminate obstetric fistulas by 2030; this can be accomplished as more women have access to competent birth attendants and as access to expert obstetric fistula repair has improved [20–22]. The WHO established a goal of fewer than 15% for failed fistula closure following repair and less than 10% for incontinence after successful fistula closure as the best range of repair outcomes for assessing the quality of services provided to patients [1].

According to studies done in the SSA, failure rates for obstetric fistula repair vary significantly depending on the context, with failure rates ranging from 11% to 58% [23–28]. These studies have outlined the rates of obstetric fistula repair failure as well as a variety of risk factors. However, the majority of these investigations are constrained by the small sample sizes, varied repair failure definitions, varied quality, and the fact that the majority of the studies were carried out at a single center in a single country, which limits the ability of these studies to generalize beyond a very specific patient population and setting. Additionally, to the best of our knowledge, there is also a limited, comprehensive analysis of the available evidence on obstetric fistula repair failure and the associated factors among women who underwent surgical repair in SSA.

Therefore, the purpose of this systematic review and meta-analysis was to determine the pooled prevalence of obstetric fistula repair failure and the associated factors among women who underwent surgical repair in SSA. This study offers a comprehensive review of the data available from existing sources to produce evidence-based information for obstetric care providers, policy planners, ministries of health, and other relevant stakeholders in SSA regarding the prevalence of obstetric fistula repair failures and associated risk factors. It also serves as a foundation for future prospective studies in the field and aims to improve the effectiveness, quality, and outcomes of fistula repair interventions in SSA.

## Materials and methods

### Protocol registration and reporting

The protocol for this systematic review and meta-analysis was developed and registered on the PROSPERO (prospective register of systematic reviews) (registration number: CRD42023409402). The Preferred Reporting Items for Systematic Review and Meta-Analysis Statement (PRISMA) guideline was used to report the review’s findings [29] (S1 File).

### Study design, setting, and search strategy/data source

To determine the pooled prevalence of obstetric fistula repair failure and associated factors among women who had repair, a systematic review and meta-analysis were carried out in SSA. The region of Africa known as SSA, which includes West Africa, Southern Africa, East Africa, and Central Africa, is situated south of the Sahara.

The studies were retrieved online using MEDLINE/PubMed, Google Scholar, Hinari, and Gray (unpublished) literature, academic archives, and manually searching references from a list of included. The search was conducted using the following keywords, either separately or in combination: prevalence, magnitude, repair failure, unsuccessful closure, successful repair with incontinence, associated factors, predictors, determinants, contributing factors, obstetric fistula, vesicovaginal fistula, rectovaginal fistula, women, female, SSA. The following keywords were utilized to obtain articles from PubMed: (Epidemiology) OR (Prevalence) OR (Magnitude) AND (Obstetric Fistula) OR (Vesico vaginal Fistula) OR (Recto vaginal Fistula) OR (Ureterovaginal Fistula) OR (Vesico uterine Fistula) AND (Unsuccessful fistula closure) OR (Incontinence following a successful closure) OR (Repair failure) AND (Associated factors) OR (Determinants) OR (Predictors AND) AND (Women who underwent obstetric fistula repair) AND (Sub-Saharan Africa) OR (SSA). Three authors (HEH, ZA, and MA) searched for articles during the months of May 1 and July 1, 2023, with no restrictions on the date of publication. Using the EndNote Version X7 reference manager, articles found through electronic searches were exported, organized, and duplicate results were removed.

### Study selection and eligibility criteria

Before retrieving the full-text articles, two researchers (HEH and BGD) independently reviewed the titles and abstracts of the chosen studies. The full-text articles were further screened using predetermined inclusion criteria. During a consensus meeting with the other reviewers (MA and ZA), disagreements regarding the final selection of studies to be included in the systematic review and meta-analysis were discussed and solved. To establish inclusion and exclusion criteria for prevalence studies, we employed the CoCoPop (Condition, Context, and Population) approach.

#### Inclusion criteria

*Participants*. Participants were women who had undergone obstetric fistula surgery or treatment.

*Study settings*. Studies carried out in SSA countries in community or institutional settings.

*Study design*. All observational studies (i.e., cross-sectional, case-control, and cohort) reporting the magnitude of obstetric fistula repair failure and its associated factors were eligible for this review.

*Publication type*. Both published (journal articles) and unpublished (master’s theses and dissertations) articles without restriction of date of publication were included.

*Language*. The review included only English-language studies.

#### Exclusion criteria

Articles addressing rape-related fistulas or those caused by non-obstetric causes (such as hysterectomy)were excluded from the study. Editorials, letters, reviews, comments, interventional studies, articles without abstracts and whose full data were not accessible despite requests from the authors, duplicate studies, and articles with poor methodological quality were excluded. Studies that only showed qualitative data on obstetric fistula repair failure in women who had repairs were also excluded.

### Measuring outcome variables

There are two main outcomes of this study. The primary outcome variable for this study, obstetric fistula repair failure, is the proportion of women with failed fistula closure and/or incontinence after successful fistula closure at 21 days following surgery. In order to determine the prevalence of obstetric fistula failure, the total number of study participants (including the sample) was divided by the number of women who experienced failed fistula closure and/or incontinence following successful fistula closure and then multiplied by 100. Identifying the determinants of obstetric fistula failure in women who have had repairs was the second outcome of this study. In order to establish the association between obstetric fistula repair failure and factors for the second outcome, we calculated the log odds ratio. Based on binary results from the primary studies, the odds ratio for significant factors was estimated.

### Data extraction

Using a pre-piloted data extraction format prepared in a Microsoft Excel spreadsheet, two authors (HEH and MA) independently extracted all essential data. All discrepancies between the two writers were settled by conversation and agreement at the time of data collection. The principal author of the original study was contacted if more details or clarifications were required. The primary author name, publication year, countries where the study was done, study setting, sample size, study design, response rate, and prevalence of obstetric fistula repair failure were all included in the data extraction form for the first outcome. For the secondary outcome (associated factors), data were retrieved in the form of two by two tables, and the log odds ratio for each factor was then calculated based on the findings of the original research.

### Assessment of the quality of the individual studies

The quality of each article chosen for review was independently evaluated by two reviewers (HEH and ZA). The methodological quality of possible studies was evaluated using the Newcastle-Ottawa scale (NOS) [30], a tool for evaluating the quality of observational studies in systematic reviews and meta-analyses, in order to determine the likelihood of bias within the included research. Study group selection was evaluated based on sample representativeness, exposure determination, sample size, and non-response rate; comparability was evaluated based on subject comparability; and outcome was evaluated based on an assessment of the outcome and a statistical test for cross-sectional studies. The NOS ratings are used to evaluate the articles’ quality and eligibility in four main categories. Category I: Selection (five points) because: ascertainment of exposure (2 points), representativeness of the sample (1 point), sampling methodology (1 point), and response rate (1 point); category II: comparability (2 points): confounding controlled (data or results adjusted for pertinent predictors, risk variables, and confounding factors (2 points); category III: outcome (3 points): assessment of outcome (2 points) and statistical tests (1 point). The next step was to calculate the quality score for each study, which for cross-sectional studies ranged from zero to ten. Finally, the meta-analysis only included high-quality publications that had a score of at least 6 out of 10. Any disagreements that arose between the reviewers were resolved through conversation or with another reviewer (BGD and MA).

### Data processing and statistical analysis

For the meta-analysis, the retrieved data were exported into STATA version 17. In order to account for the observed variability, a random-effects (DerSimonian and Laird) method was used in a meta-analysis to determine the pooled prevalence of obstetric fistula repair failure among women who had repair. An independent analysis was also done on the effects of particular determining factors on obstetric fistula repair failure. A forest plot was created and used to display the pooled effect size (prevalence and odds ratio (OR)), along with a 95% confidence interval (CI). I^2^ statistics and Cochran’s Q test were used to assess the heterogeneity between studies. The percentage of the total variation in the study estimate that is related to heterogeneity is measured using I^2^. I^2^ values vary from 0 to 100%, and those greater than 75% indicate significant study heterogeneity. A statistically significant heterogeneity was defined as having a p-value of less than 0.1. A subgroup analysis was performed with regard to the country, year of publication, and sample size in order to reduce the variance of estimated points between primary studies. Additionally, a univariate meta-regression analysis was conducted using the sample size, year of publication, and response rate as covariates.

A funnel plot was used to analyze publication bias graphically. The funnel plot’s asymmetry is an indication of possible publication bias. A p-value of less than 0.05 was used to determine the presence of significant publication bias using Egger’s test, indicating the existence of a small study effect [31]. Then, a trim and fill analysis was conducted to deal with publication bias. Moreover, sensitivity analysis was carried out to see whether individual studies had an impact on the pooled effect size. To ascertain the relationship between related factors and obstetric fistula repair failure, a log odds ratio with a 95% confidence interval (CI) was utilized. A forest plot and the odds ratio (OR) with its 95% CI were used to present the results of the meta-analysis.

## Result

### Study selection and identification

**Fig 1:**
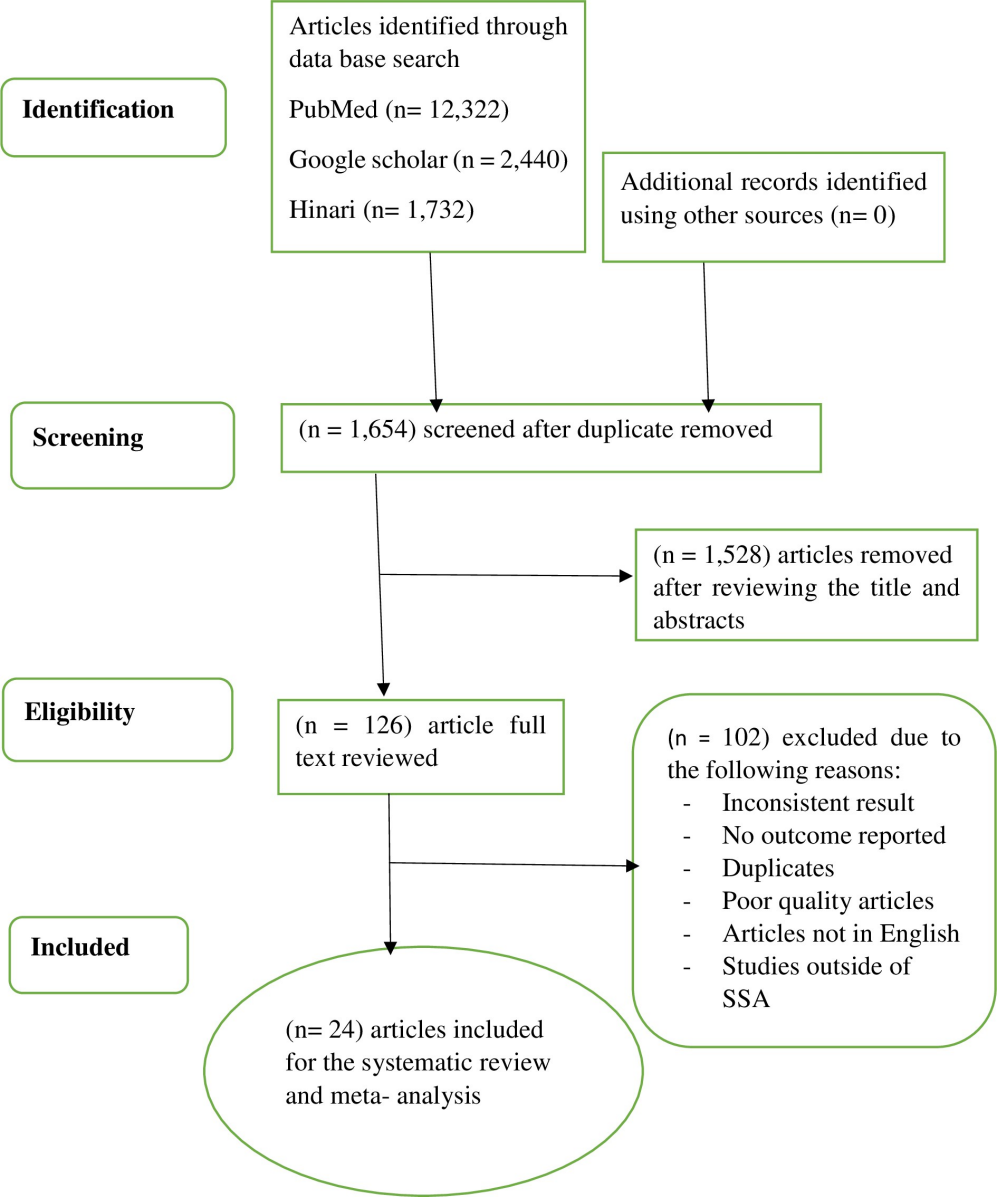
PRISMA flow diagram demonstrating the literature search and screening process.

A total of 16,504 articles were identified using an electronic database search; the manual search did not add additional studies. Of these, Endnote citation manager software version X7 deleted 14850 duplicate articles, and 1528 studies were excluded by reviewing the titles and abstracts of the remaining articles. Out of the 126 articles that were left, 102 were disregarded because they did not include the desired outcome, had inconsistent result reports, had irrelevant target groups, or had duplicated information. Finally, a total of 24 articles that satisfied the criteria for inclusion, representing 13 SSA countries, were included to the study (Fig 1).

### Characteristics and quality of the included articles

In total, 24 studies that were carried out in SSA nations were included in this meta-analysis. Regarding the year of publication, all research was published between 2007 and 2022. Thirteen articles were published during and before 2016, and eleven of the studies were published after 2016, all of which were conducted in hospitals (100%), and which took place in 13 SSA countries. In total, 9866 women who had obstetric fistulas repaired participated in the 24 studies that were included and carried out in SSA. The mean number of samples was 411, while the minimum and maximum sample sizes were 32 and 1303, respectively. In terms of study design, there were nine cohort studies, six cross-sectional studies, eight retrospective chart reviews, and one case-control study were among the included articles.

Out of the 24 articles, 5 were from Ethiopia [26, 32–35], 4 were from the Democratic Republic of the Congo [25, 36–38], 2 were from Nigeria [23, 39], 1 was from Niger [40], 2 were from Uganda [24, 41], 2 were from Zambia [16, 42], 2 were from Kenya [27, 43], 1 was from Guinea [44], 1 was from Angola [28], 1 was from Benin [45], 1 was from Rwanda [46], 1 was from Tanzania [47], and 1 was from Burkina Faso [48]. All of the included studies were published.

According to the articles that were reviewed, the prevalence of obstetric fistula repair failure ranged from the lowest 11% in Ethiopian [26], Nigerian [23], and Ugandan [24] studies to the highest 58% in an Angolan study [28].

During our quality assessment, we verified that all included studies had reliable methodological quality (NOS scores vary from 7 to 10 out of a possible 10-point total). It determined that there was moderate to almost complete agreement among investigators regarding the level of bias for the studies that were included in the final analysis (Table 1).

**Table 1:** Characteristics of the included studies for the systematic review and meta-analysis.

Authors name	Publication year	Country	Centers	Sample size	Outcome	Response rate (%)	Prevalence (%)	Quality Score
Ahmed ZA et al. [23]	2013	Nigeria	Laure fistula center	343	32	83.9	11.1	7
Aynie AA et al. [32]	2019	Ethiopia	Bahir Dar, Hamlin Fistula Center	385	136	100	35.3	10
Bello OM et al. [39]	2011	Nigeria	Ibadan, tertiary institution	155	43	77	36.1	8
Bernard L et al. [28]	2019	Angola	Centro Evangélico de Medicina do Lubango (CEML) hospital	407	234	100	58	8
Delamou A et al. [44]	2016	Guinea	Three Engender health supported repair hospitals	785	109	96	14.5	9
G. Egziabher T et al. [46]	2015	Rwanda	Public tertiary hospital	272	38	100	13.9	9
Goh GTW et al. [33]	2008	Ethiopia	Addis Ababa hospital and Bahirdar Hamlin fistula unit	1024	256	96.4	25.9	8
Hawkins L et al. [43]	2013	Kenya	Three medical centers in western Kenya	556	87	86.7	18	8
Holme A et al. [16]	2007	Zambia	Zambia’s primary fistula repair center, Monze Mission Hospital.	259	69	98.4	27.1	7
Holt L et al. [24]	2021	Uganda	Fistula Hospital in Uganda	546	59	99.1	11	9
Imakando MM et al. [42]	2022	Zambia	Managedata Teaching Hospital	32	3	56	17	7
Kayondo M et al. [41]	2011	Uganda	Mbarara Regional Referral Hospital in western Uganda	77	17	100	22.1	8
Loposso M et al. [38]	2016	Dr.Congo	Saint Luc Hospital Kisantu	166	47	100	28.3	7
Mafu MM et al. [25]	2022	Dr.Congo	Saint Joseph Hospital in Kinshasa, Panzi Hospital in Bukavu, South Kivu and HEAL Africa Hospital in Goma, North Kivu.	895	106	100	11.8	9
Magali KT et al. [47]	2016	Tanzania	Bugando Medical Centre, Mwanza	159	57	83	42.9	7
Mubikayi L et al. [37]	2017	Dr.Congo	Four fistula centers, Hospital	483	72	100	14.9	7
Mwangi HR et al. [27]	2018	Kenya	Gynocare Women’s and Fistula Hospital	357	119	88	37.9	8
Nardos R et al. [26]	2009	Ethiopia	Addis Ababa Hamlin Fistula Hospital	1303	115	81	11	8
Nsambi J et al. [36]	2019	Dr.Congo	Facilities in the south eastern part of the province of the Upper Katanga	384	66	100	17.1	9
Ouedraogo I et al. [40]	2018	Niger	Danja Fistula Center	384	177	100	46	8
Sambo BT et al. [45]	2016	Benin	Hospital of Bembereke	82	22	100	26.8	8
Sori DA et al. [34]	2016	Ethiopia	Jimma University teaching Hospital	200	26	84	15.5	7
Tadesse S et al. [35]	2022	Ethiopia	Yirgalem Hamlin fistula center	562	162	100	28.8	10
Traore TM et al. [48]	2023	Burkina Faso	regional teaching hospital	50	16	100	32	8

### Prevalence of obstetric fistula repair failure in SSA

**Fig 2:**
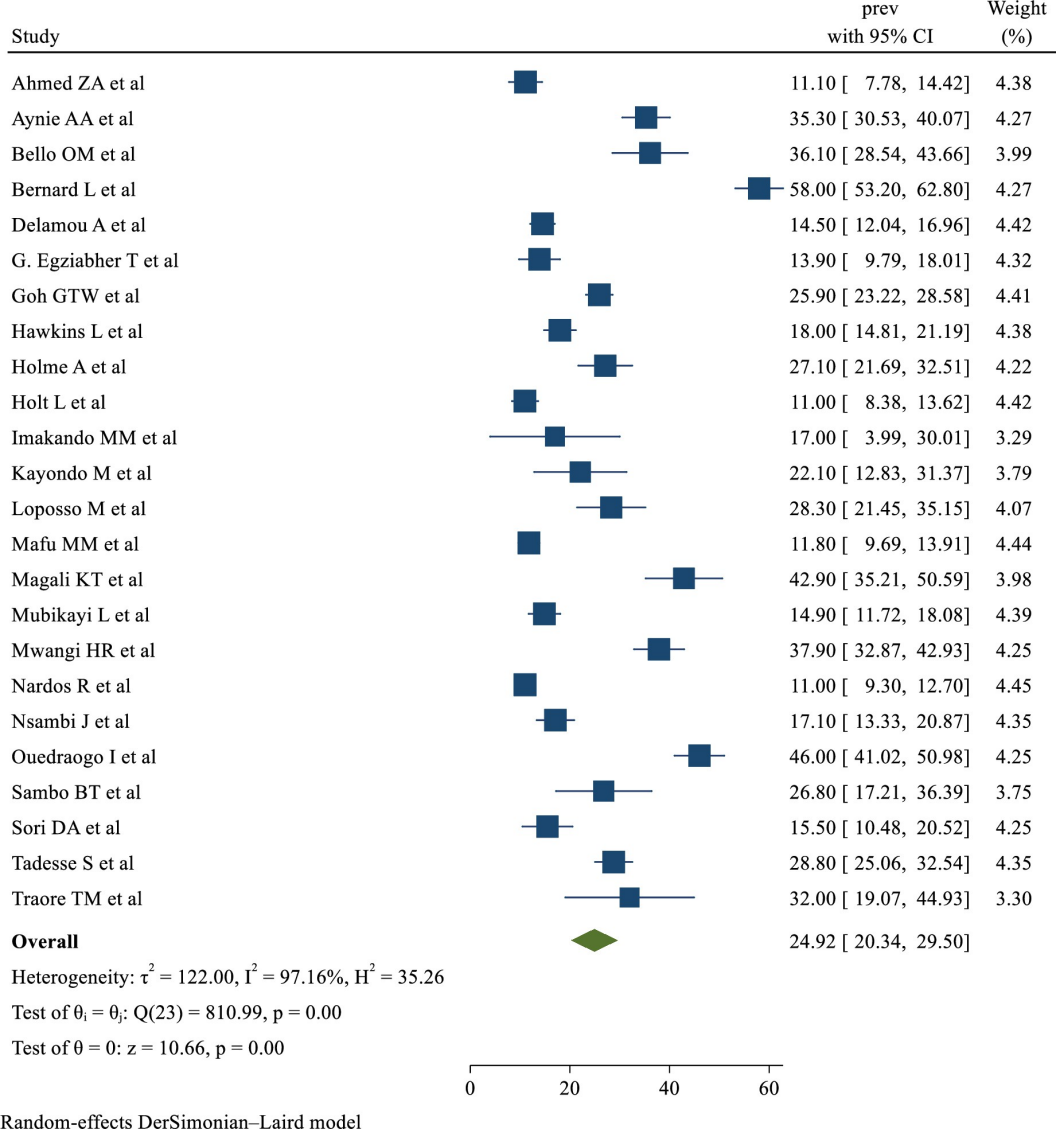
Forest plot showing the pooled prevalence of obstetric fistula repair failure in Sub-Saharan Africa. Subgroup analysis on the prevalence of obstetric fistula repair failure.

In order to assess the Der Simonian and Laird overall effect, a random-effects model was utilized because there was significant heterogeneity among the included studies (I^2^ = 97.16%, p-value < 0.001). Therefore, in SSA, the pooled prevalence of obstetric fistula repair failure among women who underwent repair was 24.92% (95% CI: 20.34–29.50%) (Fig 2).

Subgroup analysis by country, sample size, and publication year was carried out to identify the possible source of heterogeneity between studies.

**Fig 3:**
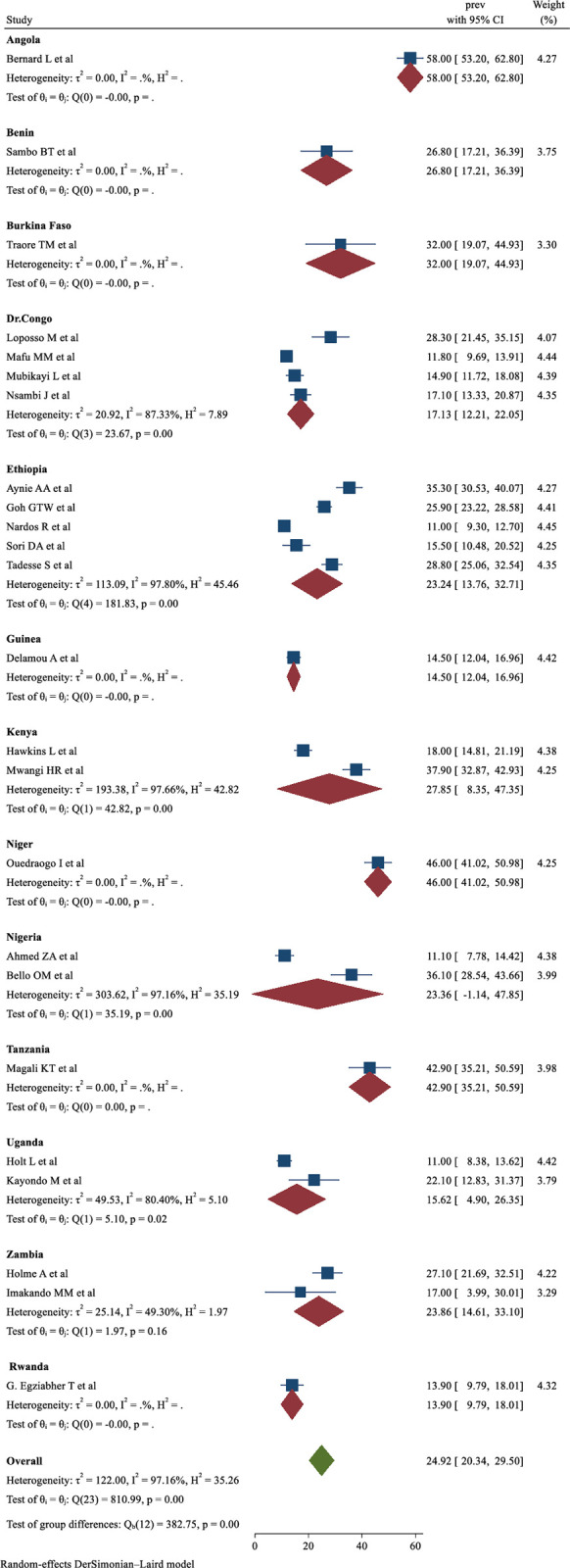
Sub-group analysis of the prevalence of obstetric fistula repair failure in Sub-Saharan Africa based on random effect model, divided by country.

On country-wise subgroup analysis, the country where the study was conducted was one of the major sources of significant heterogeneity. The highest prevalence of obstetric fistula repair failure was observed among studies conducted in Angola (58.00%, 95% CI: 53.20–62.80%) and the lowest was observed in Rwanda (13.90%, 95% CI: 9.79–18.01%) (Fig 3).

A subgroup analysis was carried out based on the sample size (mean) and publication year (mean). Accordingly, it was found that there was a 28.16% prevalence of obstetric fistula repair failure in studies that were finished after 2016 and a 28.82% prevalence in studies with a sample size of less than or equal to 411. In both cases, there was significantly higher heterogeneity among studies (Table 2).

**Table 2:** The pooled prevalence of obstetric fistula repair failure, 95% CI, and heterogeneity estimate with a p-value for the subgroup analysis.

Variables	Characteristics	Number of studies	Pooled prevalence (95%CI)	I^2^ (p-value)
Publication year	≤ 2016	13	21.91 (17.48–26.33)	94.16% (< 0.001)
	> 2016	11	28.16 (19.21–37.12)	98.24% (< 0.001)
Sample size (mean)	≤ 411	15	28.82 (20.77–36.87)	96.76% (< 0.001)
	> 411	9	18.83 (14.10–23.55)	96.45% (< 0.001)

### Publication bias

**Fig 4:**
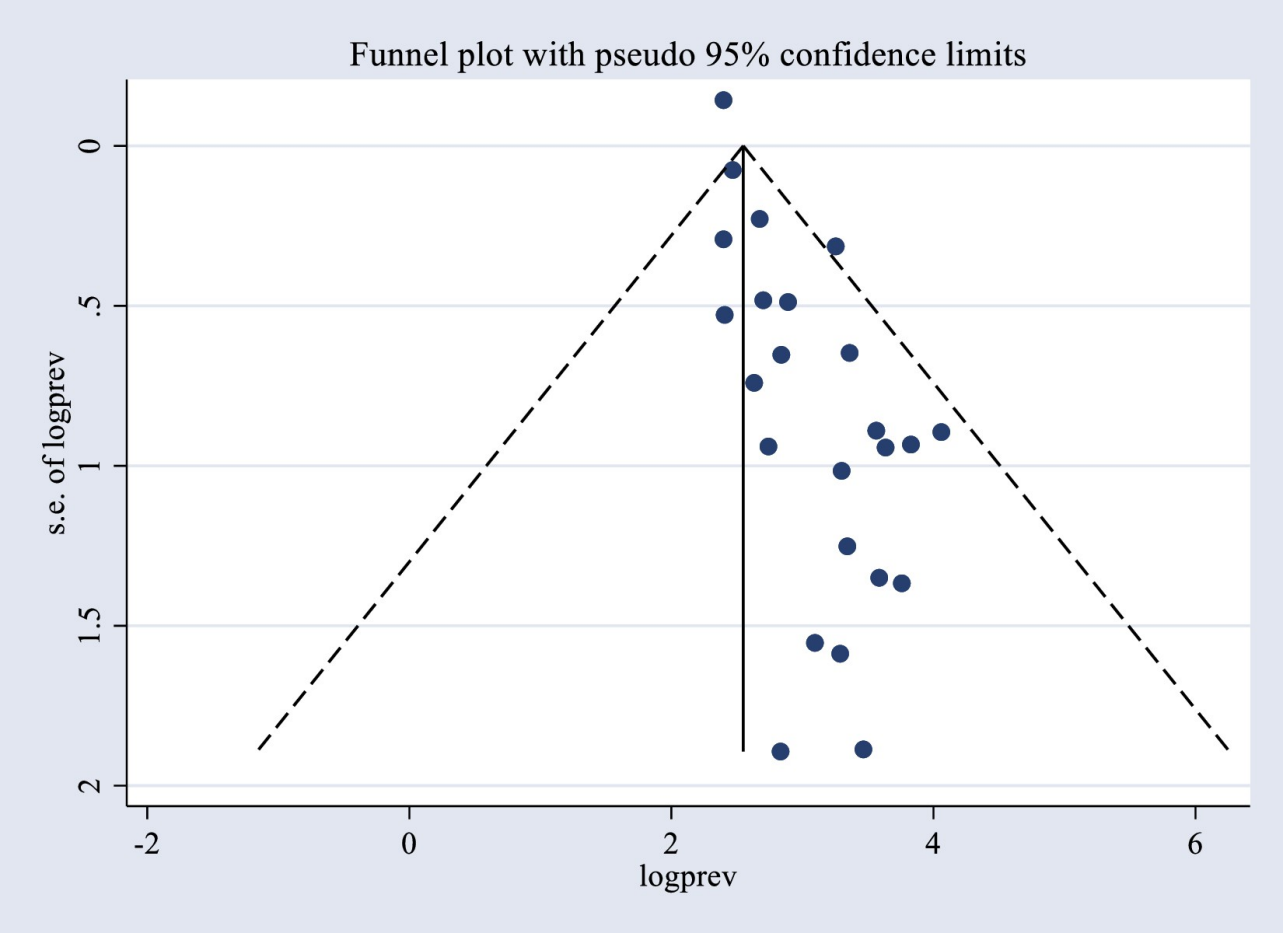
Funnel plot of publication bias for the pooled prevalence obstetric fistula repair failure and its associated factors among women who underwent repair in sub-Saharan Africa.

Egger’s tests were used to determine publication bias in the studies that were included in the meta-analysis. According to Egger’s test, there was evidence of publication bias (P-value ≤ 0.001). The nonparametric rank correlation (Begg) test also revealed there is significant publication bias (p-value of 0.021). Moreover, the funnel plot was visually examined for symmetry, and the funnel plot’s form also reveals that the asymmetric distribution of the effect estimates is a sign of publication bias (Fig 4). Therefore, the trim and fill analysis was carried out to take publication bias into account.

### Trim-and-fill analysis for the prevalence of obstetric fistula repair failure

**Fig 5:**
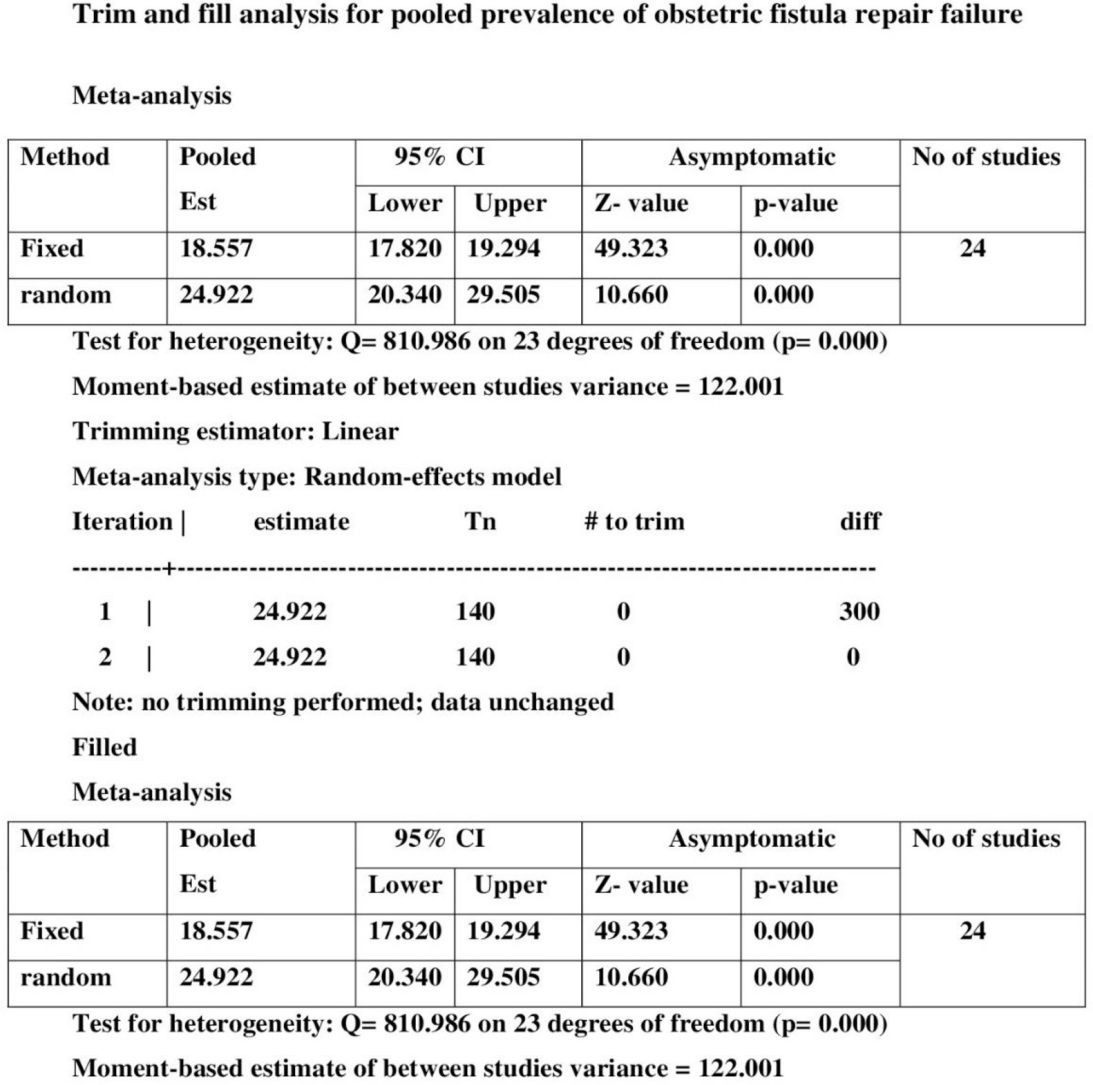
Trim-and-fill analysis for the prevalence of obstetric fistula repair failure among women who underwent surgical repair in Sub-Saharan African countries.

A trim-and-fill analysis was used to estimate the number of potential missing studies. No studies were imputed during the analysis, and no missing studies were identified. After publication bias was adjusted, the estimated pooled prevalence of obstetric fistula repair failure among women who underwent surgical repair in SSA countries appeared to be 24.92 (95% CI: 20.340–29.505%). This result is similar to the unadjusted pooled prevalence rate of obstetric fistula repair failure, with comparable degrees of heterogeneity across the studies in the random-effects model analysis (I^2^ = 97.16%, p ≤ 0.001) (Fig 5).

### Meta-regression

A univariate meta-regression was carried out utilizing sample size, publication year, and response rate as factors in order to pinpoint the potential source of heterogeneity. Only sample size, as was found using random-effects meta-regression analysis, was a significant source of heterogeneity. The chance of obstetric fistula repair failure decreased by a factor of 0.014 as the sample sizes increased (β = - 0.014 (95% CI: - 0.025–0.004), p-value = 0.007). 24.88% of the variation in overall estimates was explained by univariate meta-regression by sample size (Table 3).

**Table 3:** Univariate meta-regression analysis to determine factors related to the heterogeneity of the prevalence of obstetric fistula repair failures in Sub-Saharan African countries.

Variables	Coefficient	95% confidence interval	I^2^ (%)	R^2^ (%)	p-value
Publication year	0.311	- 0.788–1.399	97.26	0.00	0.575
Response rate	0.158	- 0.303–0.620	97.11	0.00	0.500
Sample size	- 0.014	- 0.025–0.004	96.57	24.88	0.007

### Sensitivity analysis

**Fig 6:**
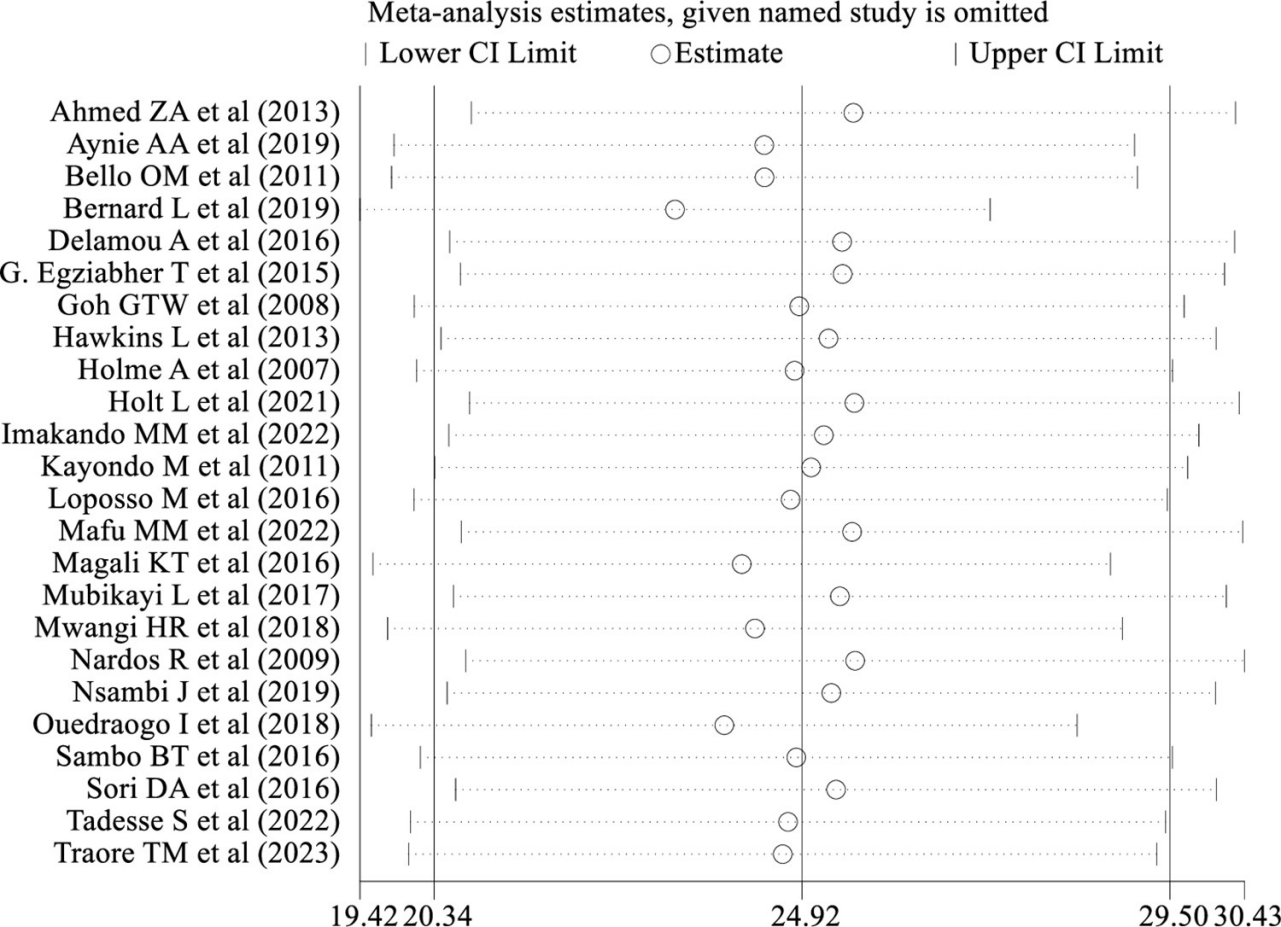
Sensitivity analysis of prevalence for each study being removed at a time: Prevalence and 95% confidence interval of obstetric fistula repair failure in Sub-Saharan Africa.

A sensitivity analysis using the random-effects model was conducted to examine the impact of individual studies on the pooled prevalence of obstetric fistula repair failure among women who underwent repair in SSA. The results showed that that no single study had a significant influence on the overall pooled prevalence of obstetric fistula repair failure among women who underwent repair (Fig 6).

### The association between urethral damage and obstetric fistula repair failure

**Fig 7:**
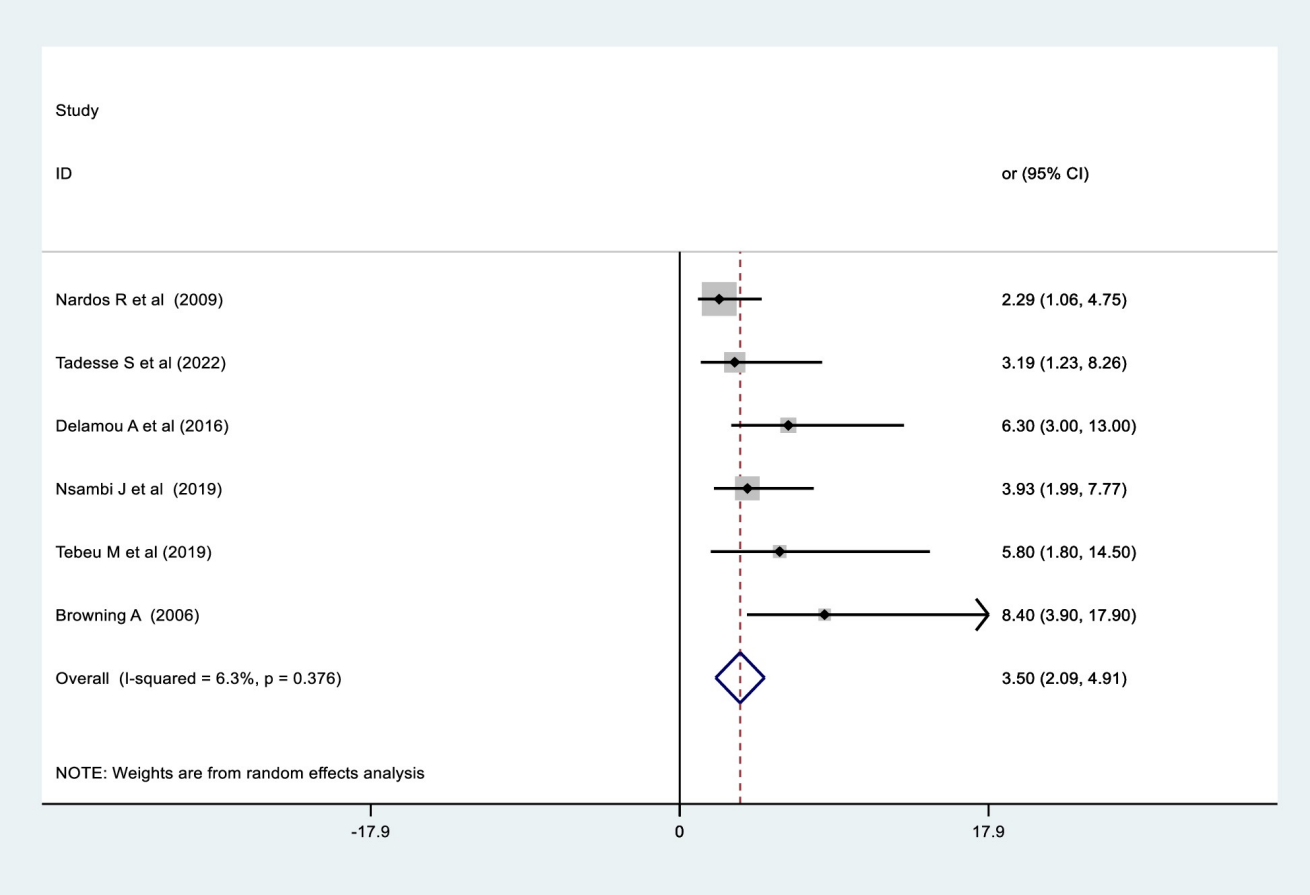
The pooled effects of complete urethral damage on obstetric fistula repair failure among women who underwent repair in Sub-Saharan Africa.

Six studies (three from Ethiopia [26, 35, 49], one from Guinea [44], one from the Democratic Republic of the Congo [36], and one from Cameron [50]) were utilised to identify the association between urethral involvement and obstetric fistula failure repair in women who underwent repair in SSA. As a result, the pooled effects of five studies using random effect meta-analysis showed that women with total urethral damage were 3.5 times more likely than those with an intact urethra to undergo obstetric fistula repair failure (OR  =  3.50, 95% CI: 2.09, 4.91) (Fig 7).

### The association between fistula size and obstetric fistula repair failure

**Fig 8:**
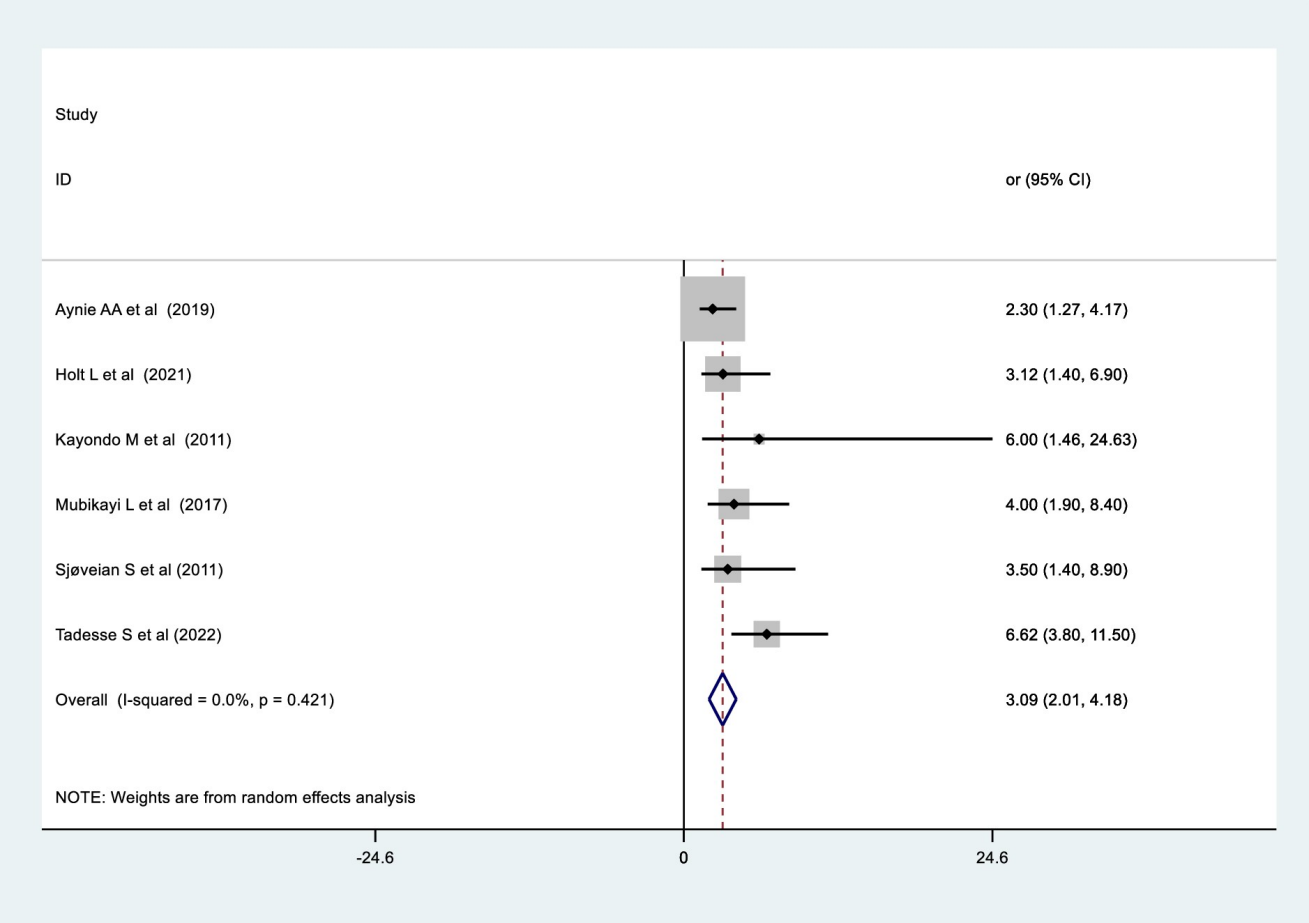
Forest plot for the association between fistula size and obstetric fistula repair failure among women who underwent repair in Sub-Saharan Africa.

The findings of six studies, two from the Democratic Republic of the Congo [37, 51], two from Ethiopia [32, 35], and two from Uganda [24, 41], respectively, showed that women with large fistula sizes (> 3 cm) were more likely to experience obstetric fistula repair failure than women with small fistula size (less than or equal to 3 cm). Our pooled data revealed that the likelihood of obstetric fistula repair failure was 3 times higher in women with fistulas larger than 3 cm than in those with fistulas smaller than or equal to 3 cm (OR = 3.09, 95% CI: 2.00,4.10), with no heterogeneity among studies (Fig 8).

### The association between duration of labor and obstetric fistula repair failure

**Fig 9:**
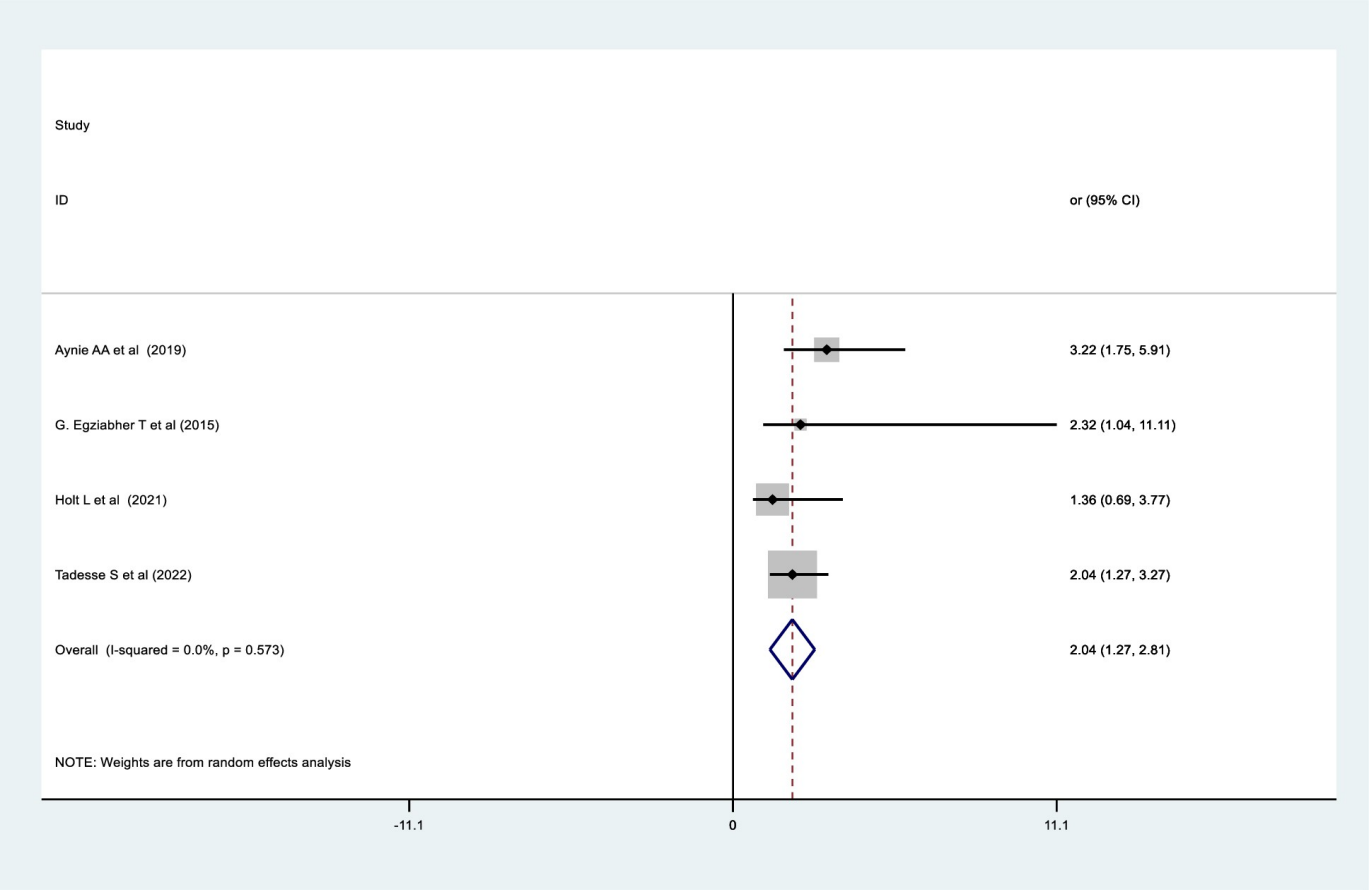
Forest plot for the association between duration of labor and obstetric fistula repair failure among women who underwent repair in Sub-Saharan Africa.

The relationship between the duration of labor and the failure of obstetric fistula repair was examined in four studies. Two studies from Ethiopia [32, 35] and one from Rwanda [46] found significant associations, but a study from Uganda [24] found no significant relationship between the two variables. According to the pooled analysis, women whose labors lasted less than or equal to 48 hours were 55% less likely to experience obstetric fistula repair failure than those whose labors were longer (> 48 hours) (OR = 2.04, 95% CI: 1.27, 2.81) (Fig 9).

### The relationship between previous fistula repair and obstetric fistula repair failure

**Fig 10:**
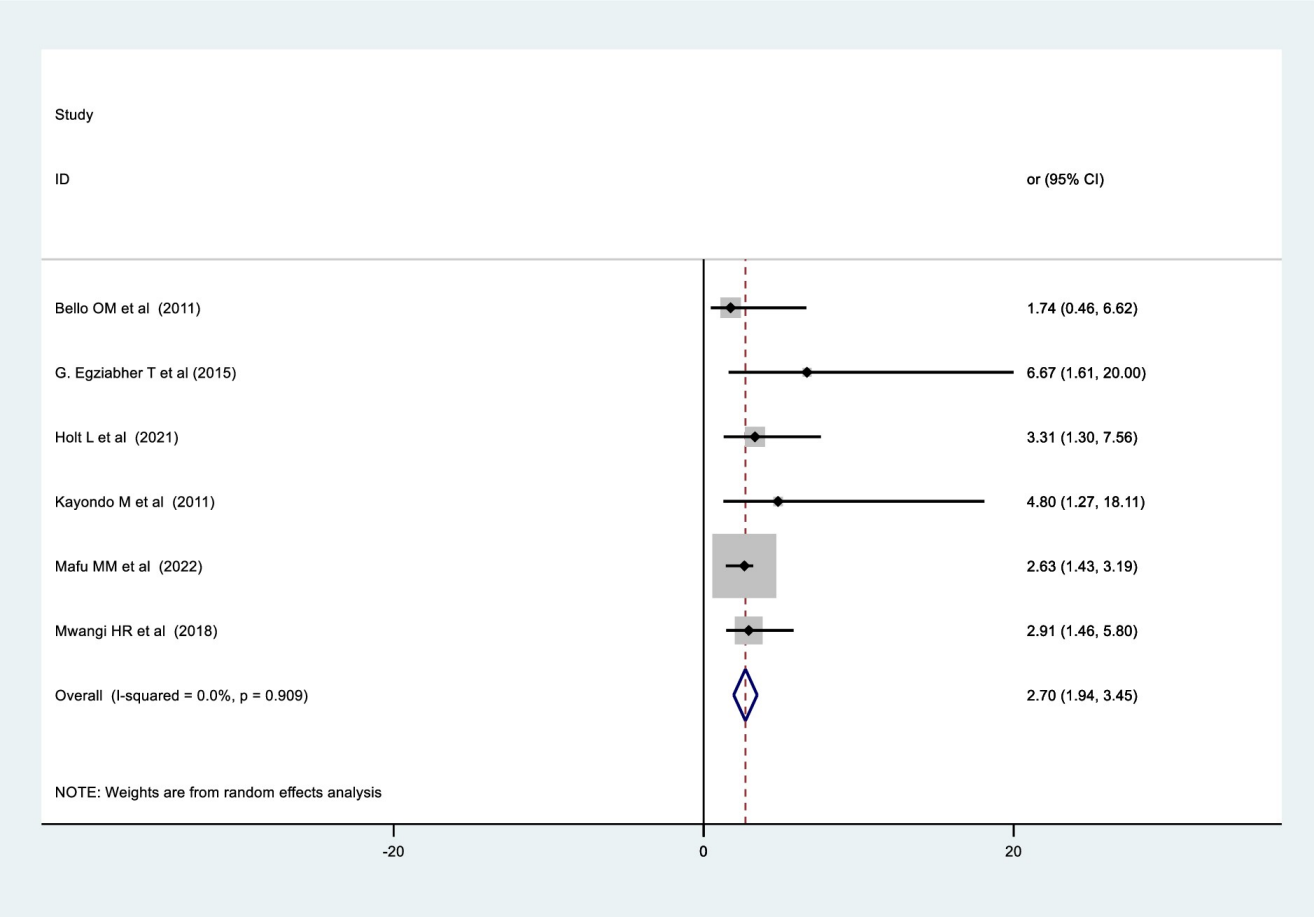
Forest plot for the relationship between previous fistula repair and obstetric fistula repair failure among who women underwent repair in Sub-Saharan Africa.

In order to determine the relationship between prior fistula repair and obstetric fistula repair failure, five studies from Uganda [24, 41], Kenya [27], Rwanda [46], and Democratic republic of the Congo [25] were identified which found a significant association between prior fistula repair and obstetric fistula repair failure. However, a single study conducted in Nigeria revealed no association between prior fistula repair and obstetric fistula repair failure [39]. The results of the pooled data showed that women who had previously had an obstetric fistula repaired had a nearly three-fold increased likelihood of having an obstetric fistula repair failure (OR = 2.70, 95% CI: 1.94 to 3.45, p-value < 0.05) (Fig 10).

### The association between postoperative complication and obstetric fistula repair failure

**Fig 11:**
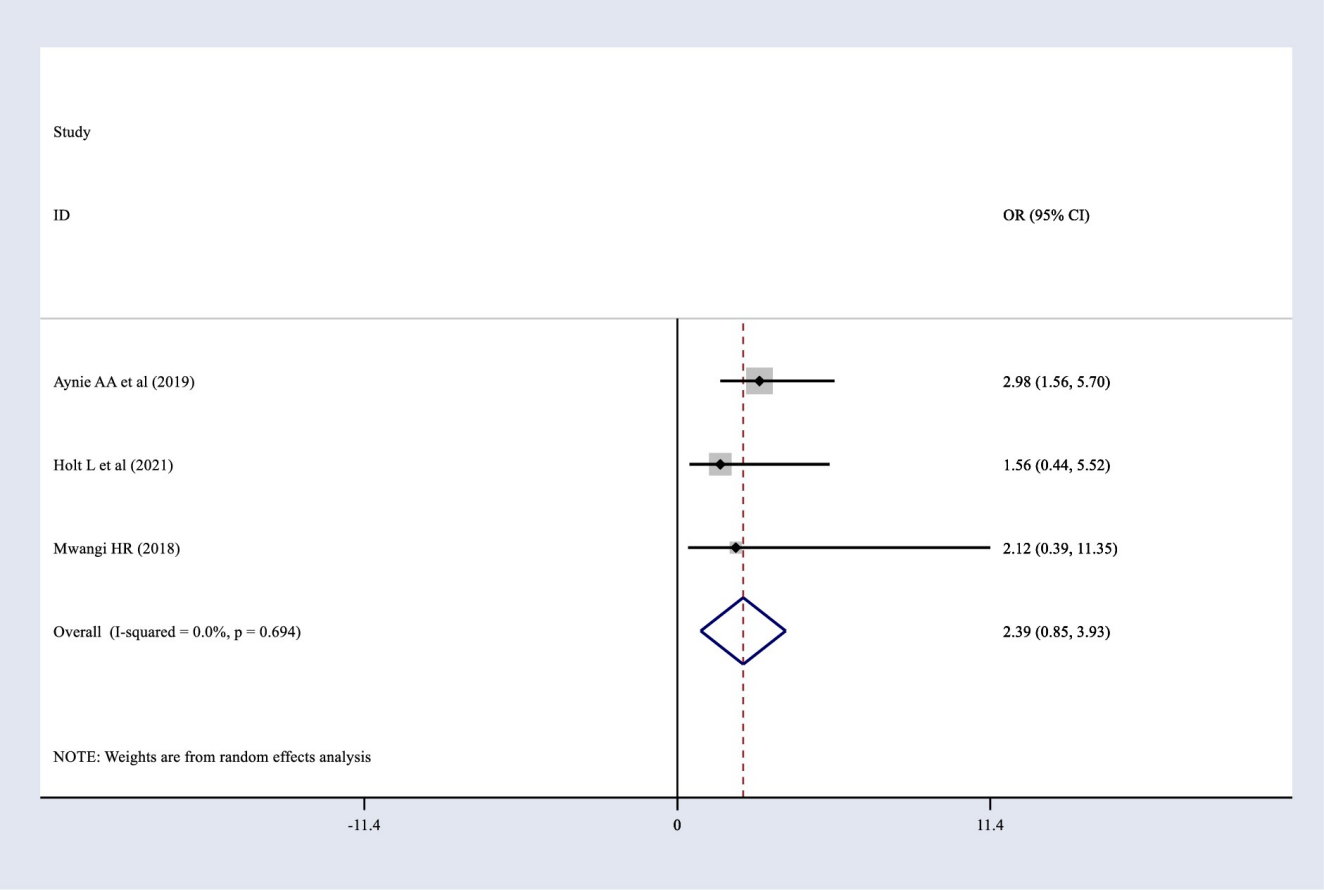
Forest plot for the association between postoperative complications and obstetric fistula repair failure among women who underwent repair in Sub-Saharan Africa.

Three studies were identified: one from Uganda, one from Kenya, and one from Ethiopia. Of the three, the Ugandan [24] and Kenyan [27] studies found no significant relationship between post-operative complications and obstetric fistula repair failure. The Ethiopian [32] study found a significant association between postoperative complications and obstetric fistula repair failure. Moreover, the pooled result showed that there is no significant association between postoperative complications and obstetric fistula repair failure (Fig 11).

## Discussion

In accordance with WHO’s Sustainable Development Goal 3.7, "by 2030, ensure universal access to sexual and reproductive health-care services, including family planning, information, and education, and the integration of reproductive health into national strategies and programs [52]." Fistula eradication by 2030 is a component of this objective [21]. In order to achieve this objective, women needed to have access to high-quality surgery [53]. Therefore, this systematic review and meta-analysis aimed to assess the pooled prevalence of obstetric fistula repair failure and its associated factors among women who underwent repair in SSA countries.

The results of this meta-analysis indicate that the pooled prevalence of obstetric fistula repair failure in SSA countries was 24.92% [95% CI: 20.34–29.50%]. This is comparable to a multicounty prospective study that revealed a nearly 20% failure rate for obstetric fistula repair [54]. These results were higher than those of a study conducted in Pakistan, which showed that 12.8% of women who underwent obstetric fistula repair experienced failure [55], and a systematic review study that revealed the failure rate after closure was 13% [56]. Additionally, it was higher than the WHO target of less than 15%, which is considered to be a sign of high-quality medical care [1]. There could be a difference in how the failure rate is defined, which could explain the discrepancy between this study’s possible differences from previous studies and the WHO target. Other possible reasons for the differences include variations in the type of repair technique, surgical expertise, and the accessibility of high-quality fistula repair services. Failure was measured, as in most studies, two weeks post-surgery [51].

Furthermore, the prevalence of obstetric fistula repair failure is strongly influenced by the patient’s age, nutritional state, and body mass index (BMI). In contrast to older patients who may have comorbidities and lower tissue quality, which can raise the chance of repair failure, younger people recover tissue more quickly and are generally in better health. Patients with a history of multiple deliveries may have severe tissue damage and scarring, which makes the repair more difficult and raises the chance of failure. Low BMI can also impact immune system function and tissue healing, increasing the risk of repair failure. Inadequate postoperative care can result in wound breakdown, infection, and other issues that can lead to repair failure. Prolonged obstructed labor during previous deliveries can also cause tissue ischemia and necrosis, which further complicates the healing process. Therefore, access to high-quality emergency care, a strong political commitment to effective fistula care, infection prevention, postoperative monitoring, sufficient preoperative nutrition, and BMI optimization are crucial for successful obstetric fistula repair.

The results of the sub-group analysis showed that there were variations in the pooled prevalence of obstetric fistula repair failure among the SSA countries, with Angola having the highest prevalence of 58% [28], followed by a study in Burkina Faso [48], and Rwanda having the lowest prevalence of 13.9% [46]. There could be several reasons for the variations in the rate of obstetric fistula repair failure among the SSA countries, including variations in the accessibility of facilities equipped with the necessary tools and repair techniques, surgical experience and training of the surgeons, fistula characteristics, nutritional status, post-operative nursing care, multiparty, and the presence of comorbid infections. Therefore, implementing interventions suitable for each country is essential to rise the success rate of obstetric fistula repair, particularly in assessing women’s nutritional status, any presence of co-morbidities, and enhancing pre- and post-operative care to reduce complications following obstetric fistula repair.

Women with total urethral damage were more likely than those with an intact urethra to experience obstetric fistula repair failure. This finding is in line with a systematic review study [57] and a multicounty prospective study [54]. The finding might be related to the fact that most women who give birth vaginally, have prolonged labors, and have had multiple deliveries are more likely to have a fistula that extends to the urethra. Urethral involvement can also affect bladder size and sphincter mechanisms, which can make surgical repair more difficult. Persistent irritation of the wound site from urine incontinence may also raise the risk of repair failure [54]. As a result, for women who benefit from a Caesarean section (CS), the extension of the fistula that involves the urethra is limited compared to those who still deliver vaginally, which might also reduce the problem encountered in prolonged labor.

The likelihood of obstetric fistula repair failure was higher among women with big fistula sizes (> 3 cm) than among those with smaller or equal to 3 cm fistula sizes. This finding was in line with a study on factors determining recurrence after vesicovaginal fistula repair that was undertaken in Pakistan [55] and a systematic review study [58]. However, a multicounty prospective study indicated that there is no relationship between the size of the fistula and obstetric fistula repair failure [54]. The findings might be explained by the larger fistula’s potential for difficulty in fully mobilizing to permit a tension-free repair, which could make the procedure more challenging and complex. It may also be related to the idea that the greater the wound edge, the higher the risk of infection and dehiscence [49, 59]. Due to the increased tissue damage and complexity of the treatments involved, larger fistulas have a worse success rate for fistula repair. For repairs to be successful, early discovery and prompt intervention are essential. Addressing this relationship calls for a multidisciplinary strategy that involves specialist surgical knowledge, thorough pre- and post-operative care, and initiatives to provide timely access to obstetric services. By addressing the specific challenges associated with large fistulas, it is possible to improve surgical outcomes and ultimately reduce the burden of obstetric fistulas for affected women.

Women with a history of prior obstetric fistula repair were at a higher risk of experiencing repair failure. These results are consistent with multicounty prospective research [54] and a systematic review study [58]. The findings might be explained by the fact that repeated attempts to close an obstetric fistula result in more vaginal scarring, further harm to healthy tissues, and a foreign body reaction to the stitches, all of which increase the risk of repair failure and decrease the likelihood of regaining physiological function. As a result, women who have experienced several repairs can be more susceptible to health issues such as infection, pain, erectile dysfunction, and secondary infertility [58]. The study emphasizes the importance of selecting operable patients, hiring skilled surgeons, optimizing fistula repair outcomes, and developing national policies. It recommends extended hospital stays for women, scheduling follow-up sessions, and promoting post-repair care [59].

Moreover, women with prolonged labor (> 48 hours) were associated with a higher risk of experiencing obstetric fistula repair failure. The finding could be explained by the fact that ischemia necrosis of the soft tissues around the bladder, rectum, and/or vagina results in tissue damage and associated scarring, which gets worse with the length of labor and ultimately causes repair failure [33, 59]. Furthermore, inadequate nutrition, restricted access to healthcare, and postponing seeking medical attention are among the additional risk factors for fistula repair failure that are frequently linked to prolonged labor. These elements may raise the risk of a fistula recurrence and lead to poor surgical results. Obstetric fistulas can have negative psychological and emotional effects in addition to physical ones, which may affect the success of repair surgeries. Improving access to prompt and expert obstetric care, addressing underlying social determinants of health, and offering comprehensive support to women undergoing fistula repair surgeries are all necessary components of a comprehensive strategy to address the link between prolonged labor and obstetric fistula repair failure.

Despite the non-significant association between post-operative complications and obstetric fistula repair failure in this meta-analysis, women who experienced postoperative complications or infections were more likely than their counterparts to encounter fistula repair failure. The quality of services, especially the quality of surgery, is likely to be related to complications such as urinary retention, wound infection, and bleeding. Furthermore, healing might be impaired by an infection at the repair site [60]. Therefore, implementing infection prevention strategies (IPS) or providing postoperative care together with regular patient monitoring at each visit may help lower the likelihood of obstetric fistula repair failure.

### Implication for clinical practice

The study highlights the pressing need to enhance fistula repair programs and address the high prevalence of obstetric fistula in SSA. In order to lower the fistula repair failure rate and enhance the quality of life for women, country-specific interventions are required. The study points out the potential and gaps in fistula repair services and emphasizes how crucial it is to fund women’s empowerment, health, and rights in order to achieve the Sustainable Development Goals (SDG) [52].

The development of surgical techniques, instruments, and infrastructure is essential for surgical success. Specifically, the study emphasizes the significance of a multi-disciplinary to patient management by evaluating the effectiveness of preoperative and postoperative treatment and assessing women’s nutritional status, maternal age, co-morbidity, and obstructed labor as critical risk factors associated with repair failure in women with obstetric fistulas. For obstetric fistula repair to be successful, these issues must be addressed through preoperative optimization, careful surgical technique, and comprehensive postoperative care. It also suggests incorporating these findings into clinical practices to improve women’s quality of life and contribute to the global literature.

### Limitation of the study

Only English-language articles were taken into consideration for this systematic review and meta-analysis. In addition, the majority of the studies included in the analysis had small sample sizes, which could have an impact on the pooled results. Only 13 SSA countries were represented; as a result, conducting a meta-analysis with a small number of articles reduces statistical power, allows for large standard errors. The causes indicated above led to the discovery of publication bias in this meta-analysis. Only 13 SSA countries were represented in the included research; all of the SSA countries may be underrepresented, due to their poor quality, inadequate description of the outcome variable, and inaccessibility of the full-text article despite repeated requests to the corresponding authors of the original research. Furthermore, as most research reports repair outcomes using different definitions, comparisons of obstetric fistula repair failures become challenging. Others describe obstetric fistula failure as a combination of failed fistula closure and continence after repair surgery, while others merely show the overall rates of fistula closure.

## Conclusion and recommendations

Women who received surgical treatment for obstetric fistulas in SSA countries experienced more repair failures than what the WHO recommends. Obstetric fistula repair failure was affected by status of urethral damage, fistula size, duration of labor, types of fistula, and history of previous repairs.

Therefore, policymakers and health care organizations that focus on women’s health should prioritize reducing obstetric fistula repair failure and preventing all risk factors (poor nutrition, multiparty, obstructed labor, and maternal age) that lead to conditions like large fistulas, urethral damage, and repeat repair. As a result, country-specific interventions were recommended to improve the application and effectiveness of fistula care initiatives as well as the standard of services at repair facilities.

Expanding sufficient facilities that offers pre- and post-operative care and outfitted with trained surgeons is also essential. These facilities should also handle patient transportation concerns, assess women’s nutritional status, and co-morbidity, and show a strong political commitment to efficient fistula care.

Moreover, the current study’s findings will contribute to the body of global literature and provide guidance for future research. Specifically, we advise that future investigations combine all studies that have been published in both English and non-English languages and include all qualitative studies in order to more precisely identify the extent of obstetric fistula repair failure in SSA.

## Supporting information

S1 File PRISMA (Preferred Reporting Items for Systematic review and Meta-Analysis) 2020 checklist: An updated guideline for reporting systematic reviews. Recommended items addressed in our systematic review and meta-analysis.
(DOCX)

S2 File Dataset on obstetric fistula repair failure among women who underwent repair in Sub-Saharan Africa. (XLSX)

## Abbreviation

BMI: Body Mass Index
CoCoPop: Condition, Context, Population
CI: Confidence Interval
IPS: Infection Prevention Strategies
NOS: Newcastle Ottawa Scale
OR: Odds Ratio
SDG: Sustainable Development Goals
UN: United Nations
PROSPERO: Prospective Register of Systematic Reviews
PRISMA: Preferred Reporting Items for Systematic Reviews and Meta-Analyses
SSA: Sub-Saharan Africa
SDG: Sustainable Development Goals
WHO: World Health Organization

## References

[1] De Bernis L (2007) Obstetric fistula: guiding principles for clinical management and programme development, a new WHO guideline. International Journal of Gynecology & Obstetrics 99: S117–S121. doi: 10.1016/j.ijgo.2007.06.032 17880979

[2] Wall LL, Arrowsmith SD, Briggs ND, Browning A, Lassey A (2005) The obstetric vesicovaginal fistula in the developing world. Obstetrical & gynecological survey 60: S3–S51. doi: 10.1097/00006254-200507001-00002 16034313

[3] Tafesse B, Muleta M, Michael AW, Aytenfesu H (2006) Obstetric fistula and its physical, social and psychological dimension: The Etiopian scenario. Acta Urol 23: 25–31.

[4] Baker Z, Bellows B, Bach R, Warren C (2017) Barriers to obstetric fistula treatment in low‐income countries: a systematic review. Tropical Medicine & International Health 22: 938–959. doi: 10.1111/tmi.12893 28510988

[5] Swain D, Parida SP, Jena SK, Das M, Das H (2020) Prevalence and risk factors of obstetric fistula: implementation of a need-based preventive action plan in a South-eastern rural community of India. BMC women’s health 20: 1–10. 32131799 10.1186/s12905-020-00906-w PMC7055058

[6] den Hollander GC, Janszen EW (2020) Obstetric fistulas in Uganda: scoping review using a determinant of health approach to provide a framework for health policy improvement. BMC pregnancy and childbirth 20: 1–8. 10.1186/s12884-020-02951-7 PMC7189698 32349703

[7] WHO (2018) Obstetric fistula. WHO.

[8] Wall LL (2006) Obstetric vesicovaginal fistula as an international public-health problem. The Lancet 368: 1201–1209. doi: 10.1016/S0140-6736(06)69476-2 17011947

[9] Treuthart MP (2015) No Woman, No Cry-Ending the War on Women Worldwide and the International VIolence against Women Act (I-VAWA). BU Int’l LJ 33: 73.

[10] Ahmed S, Holtz S (2007) Social and economic consequences of obstetric fistula: life changed forever? International Journal of Gynecology & Obstetrics 99: S10–S15. doi: 10.1016/j.ijgo.2007.06.011 17727854

[11] Wall LL (1996) Obstetric fistulas in Africa and the developing world: new efforts to solve an age-old problem. Women’s Health issues 6: 229–234. doi: 10.1016/1049-3867(96)00004-7 8754673

[12] Karateke A, Cam C, Ozdemir A, Guney B, Vatansever D, et al. (2010) Original research Characteristics of obstetric fistulas and the need for a prognostic classification system. Archives of Medical Science 6: 253–256. 22371755 10.5114/aoms.2010.13904 PMC3281348

[13] Muleta M, Hamlin EC, Fantahun M, Kennedy RC, Tafesse B (2008) Health and social problems encountered by treated and untreated obstetric fistula patients in rural Ethiopia. Journal of Obstetrics and Gynaecology Canada 30: 44–50. doi: 10.1016/S1701-2163(16)32712-8 18198067

[14] Wall LL, Karshima JA, Kirschner C, Arrowsmith SD (2004) The obstetric vesicovaginal fistula: characteristics of 899 patients from Jos, Nigeria. American journal of obstetrics and gynecology 190: 1011–1016. doi: 10.1016/j.ajog.2004.02.007 15118632

[15] Browning A, Fentahun W, Goh JTW (2007) The impact of surgical treatment on the mental health of women with obstetric fistula. BJOG: An International Journal of Obstetrics & Gynaecology 114: 1439–1441. doi: 10.1111/j.1471-0528.2007.01419.x 17903234

[16] Holme A, Breen M, MacArthur C (2007) Obstetric fistulae: a study of women managed at the Monze Mission Hospital, Zambia. BJOG: An International Journal of Obstetrics & Gynaecology 114: 1010–1017. 17506793 10.1111/j.1471-0528.2007.01353.x

[17] Goh JT, Sloane KM, Krause HG, Browning A, Akhter S (2005) Mental health screening in women with genital tract fistulae. BJOG: An International Journal of Obstetrics & Gynaecology 112: 1328–1330. doi: 10.1111/j.1471-0528.2005.00712.x 16101616

[18] Roush KM (2009) Social implications of obstetric fistula: an integrative review. Journal of midwifery & women’s health 54: e21–e33. doi: 10.1016/j.jmwh.2008.09.005 19249652

[19] Nnamuchi O, Ezike E, Odinkonigbo J (2016) Obstetric Fistula-A Menace to Mental Health: Does Fidelity to Country Obligations under the Millennium Development Goals and Human Rights Regimes Provide an Antidote. Annals Health L 25: 65.

[20] Drew LB (2022) A Human Rights Approach Toward Eradicating Obstetric Fistula: Expanding Data Collection, Prevention, Treatment, and Continuing Support for Women and Girls Who Have Been Neglected. A Multidisciplinary Approach to Obstetric Fistula in Africa: Public Health, Anthropological, and Medical Perspectives: Springer. pp. 7–23.

[21] Slinger G, Trautvetter L (2020) Addressing the fistula treatment gap and rising to the 2030 challenge. International Journal of Gynecology & Obstetrics 148: 9–15. 10.1002/ijgo.13033 PMC7004198 31943185

[22] Anastasi E, Asiamah B, Lal G (2020) Leaving no one behind: Is the achievement of the Sustainable Development Goals possible without securing the dignity, rights, and well‐being of those who are “invisible”? International Journal of Gynecology & Obstetrics 148: 3–5. 10.1002/ijgo.13031 PMC7004130 31943179

[23] Ahmed ZD, Abdullahi HM, Yola AI, Yakasai IA (2013) Obstetrics fistula repairs in Kano, Northern Nigeria: The journey so far. Annals of Tropical Medicine & Public Health 6.

[24] Holt L, Potluri T, Tanner JP, Duffy S, Wasingya L, et al. (2021) Risk factors for early and late failures following repair of urogenital fistulas. International Urogynecology Journal: 1–10. doi: 10.1007/s00192-020-04606-9 33416963

[25] Mafu MM, Banze DFK, Aussak BTT, Kolié D, Camara BS, et al. (2022) Factors associated with surgical repair success of female genital fistula in the Democratic Republic of Congo: Experiences of the Fistula Care Plus Project, 2017–2019. Tropical Medicine & International Health 27: 831–839. doi: 10.1111/tmi.13794 35749231 PMC9541372

[26] Nardos R, Browning A, Chen CCG (2009) Risk factors that predict failure after vaginal repair of obstetric vesicovaginal fistulae. American journal of obstetrics and gynecology 200: 578. e571–578. e574. doi: 10.1016/j.ajog.2008.12.008 19200932

[27] Mwangi H, wang’ombe A, Mabeya H, Kipruto H, Wanjala A (2018) Factors associated with obstetric fistula repair failure among women admitted at Gynocare Women’s and Fistula Hospital in Kenya, 2012–2016: a case control study. Nepal Journal of Obstetrics and Gynaecology 13.

[28] Bernard L, Giles A, Fabiano S, Giles S, Hudgins S, et al. (2019) Predictors of obstetric fistula repair outcomes in Lubango, Angola. Journal of Obstetrics and Gynaecology Canada 41: 1726–1733. doi: 10.1016/j.jogc.2019.01.025 30987849

[29] Moher D, Liberati A, Tetzlaff J, Altman DG, PRISMA Group* t (2009) Preferred reporting items for systematic reviews and meta-analyses: the PRISMA statement. Annals of internal medicine 151: 264–269. doi: 10.7326/0003-4819-151-4-200908180-00135 19622511

[30] Peterson J, Welch V, Losos M, Tugwell P (2011) The Newcastle-Ottawa scale (NOS) for assessing the quality of nonrandomised studies in meta-analyses. Ottawa: Ottawa Hospital Research Institute 2: 1–12.

[31] Lin L, Chu H, Murad MH, Hong C, Qu Z, et al. (2018) Empirical Comparison of Publication Bias Tests in Meta-Analysis. Journal of General Internal Medicine 33: 1260–1267. doi: 10.1007/s11606-018-4425-7 29663281 PMC6082203

[32] Aynie AA, Yihunie AG, Munae AM (2019) Magnitude of repair failure and associated factors among women undergone obstetric fistula repair in Bahir Dar Hamlin Fistula Center, Amhara Region, Northwest Ethiopia.

[33] Goh JTW, Browning A, Berhan B, Chang A (2008) Predicting the risk of failure of closure of obstetric fistula and residual urinary incontinence using a classification system. International Urogynecology Journal 19: 1659–1662. doi: 10.1007/s00192-008-0693-9 18690403

[34] Sori DA, Azale AW, Gemeda DH (2016) Characteristics and repair outcome of patients with Vesicovaginal fistula managed in Jimma University teaching Hospital, Ethiopia. BMC Urology 16: 41. doi: 10.1186/s12894-016-0152-8 27406310 PMC4942998

[35] Tadesse S, Ejigu N, Edosa D, Ashegu T, Dulla D (2022) Obstetric fistula repair failure and its associated factors among women underwent repair in Yirgalem Hamlin fistula center, Sidama Regional State, Southern Ethiopia, 2021: a retrospective cross sectional study. BMC Women’s Health 22: 288. doi: 10.1186/s12905-022-01866-z 35811314 PMC9272558

[36] Nsambi J, Mukuku O, Kakudji P, Kakoma J-B (2019) Model predicting failure in surgical repair of obstetric vesicovaginal fistula. The Pan African Medical Journal 34: 91–91. 31934234 10.11604/pamj.2019.34.91.20547 PMC6945673

[37] Mubikayi L, Matson DO, Lokomba V, Mboloko J, Kamba JP, et al. (2017) Determinants of outcomes and prognosis score in obstetric vesico-vaginal fistula repair. Open Journal of Obstetrics and Gynecology 7: 767.

[38] Loposso M, Hakim L, Ndundu J, Lufuma S, Punga A, et al. (2016) Predictors of recurrence and successful treatment following obstetric fistula surgery. Urology 97: 80–85. doi: 10.1016/j.urology.2016.03.079 27496296

[39] Morhason-Bello I, Ojengbede O, Adedokun B, Oladokun A, Okunlola M (2011) Obstetric fistulae repair in a Nigerian Tertiary Health Institution; Lessons learnt from the outcome of care. Tropical Journal of Obstetrics and Gynaecology 28: 122–128.

[40] Ouedraogo I, Payne C, Nardos R, Adelman AJ, Wall LL (2018) Obstetric fistula in Niger: 6-month postoperative follow-up of 384 patients from the Danja Fistula Center. International urogynecology journal 29: 345–351. doi: 10.1007/s00192-017-3375-7 28600757 PMC5847061

[41] Kayondo M, Wasswa S, Kabakyenga J, Mukiibi N, Senkungu J, et al. (2011) Predictors and outcome of surgical repair of obstetric fistula at a regional referral hospital, Mbarara, western Uganda. BMC urology 11: 1–9. 22151960 10.1186/1471-2490-11-23 PMC3252285

[42] Imakando MM, Michelo C, Mkandawire T, Kasonka L (2022) Characteristics and Surgical Repair Outcomes of Obstetric Fistula Patients Managedata Teaching Hospital in Zambia: A Retrospective Cross-Sectional Study. Med j Zambia: 146–156.

[43] Hawkins L, Spitzer RF, Christoffersen-Deb A, Leah J, Mabeya H (2013) Characteristics and surgical success of patients presenting for repair of obstetric fistula in western Kenya. International Journal of Gynecology & Obstetrics 120: 178–182. doi: 10.1016/j.ijgo.2012.08.014 23141371

[44] Delamou A, Delvaux T, Beavogui AH, Toure A, Kolié D, et al. (2016) Factors associated with the failure of obstetric fistula repair in Guinea: implications for practice. Reproductive Health 13: 1–9. 27821123 10.1186/s12978-016-0248-3 PMC5100224

[45] Sambo B, Missikpode C, Salifou K, Hodonou A, Mensah E (2016) Developing a simple proof of concept clinical decision-making tool for predicting surgical outcomes after obstetric fistula repair in a developing country. Tropical and Medicine Surgery 4: 1–7.

[46] Egziabher TG, Eugene N, Ben K, Fredrick K (2015) Obstetric fistula management and predictors of successful closure among women attending a public tertiary hospital in Rwanda: a retrospective review of records. BMC research notes 8: 1–7. 26654111 10.1186/s13104-015-1771-y PMC4676892

[47] Magali KT, Seni J, Massinde AN, Gumodoka B, Rumanyika RN (2017) SHORT-TERM COMPLICATIONS AND ASSOCIATED FACTORS AMONG WOMEN UNDERGOING OBSTETRIC FISTULA REPAIR AT BUGANDO MEDICAL CENTRE, MWANZA, TANZANIA. Ethiopian Medical Journal 55.

[48] Traore TM, Ouedraogo S, Kabore M, Ouedraogo S, Traore JJ (2023) Characteristics of obstetric urogenital fistulas in a regional teaching hospital in Burkina Faso: a retrospective cross-sectional study. The Pan African Medical Journal 44. 10.11604/pamj.2023.44.105.35733 PMC10219834 37250682

[49] Browning A (2006) Risk factors for developing residual urinary incontinence after obstetric fistula repair. BJOG: An International Journal of Obstetrics & Gynaecology 113: 482–485. doi: 10.1111/j.1471-0528.2006.00875.x 16489933

[50] Tebeu Marie P, Ekono M, Noa Ndoua C, Meutchi G, Mawamba Y, et al. (2019) Surgical outcome of genito-urinary obstetric fistulas (GUOF) with or without bladder neck involvement: an experience from the University Teaching Hospital, Yaoundé, Cameroon. Obstet Gynecol Int J 10: 223–228.

[51] Sjøveian S, Vangen S, Mukwege D, Onsrud M (2011) Surgical outcome of obstetric fistula: a retrospective analysis of 595 patients. Acta obstetricia et gynecologica Scandinavica 90: 753–760. doi: 10.1111/j.1600-0412.2011.01162.x 21542810

[52] Organization WH Sustainable Development Goals (SDGs).

[53] Rane A, Browning A, Majinge P, Pope R (2020) Challenges in the field of obstetric fistula. International Journal of Gynecology & Obstetrics 148: 6–8. doi: 10.1002/ijgo.13032 31943187 PMC7004184

[54] Barone MA, Frajzyngier V, Ruminjo J, Asiimwe F, Barry TH, et al. (2012) Determinants of postoperative outcomes of female genital fistula repair surgery. Obstet Gynecol 120: 524–531. doi: 10.1097/AOG.0b013e31826579e8 22914460 PMC3437437

[55] AJaved A (2015) Doctor! Will I be dry? Factors determining recurrence after vesicovaginal fistula repair. JPMA: Journal of Pakistan Medical Association 65: 954. 26338740

[56] Hillary CJ, Osman NI, Hilton P, Chapple CR (2016) The Aetiology, Treatment, and Outcome of Urogenital Fistulae Managed in Well- and Low-resourced Countries: A Systematic Review. European Urology 70: 478–492. doi: 10.1016/j.eururo.2016.02.015 26922407

[57] Frajzyngier V, Ruminjo J, Barone MA (2012) Factors influencing urinary fistula repair outcomes in developing countries: a systematic review. Am J Obstet Gynecol 207: 248–258. doi: 10.1016/j.ajog.2012.02.006 22475385 PMC3398205

[58] Gutman R, Dodson J, Mostwin J (2007) Complications of treatment of obstetric fistula in the developing world: gynatresia, urinary incontinence, and urinary diversion. International Journal of Gynecology & Obstetrics 99: S57–S64. doi: 10.1016/j.ijgo.2007.06.027 17803995

[59] McFadden E, Taleski SJ, Bocking A, Spitzer RF, Mabeya H (2011) Retrospective Review of Predisposing Factors and Surgical Outcomes in Obstetric Fistula Patients at a Single Teaching Hospital in Western Kenya. Journal of Obstetrics and Gynaecology Canada 33: 30–35. doi: 10.1016/S1701-2163(16)34769-7 21272433

[60] Barone MA, Widmer M, Arrowsmith S, Ruminjo J, Seuc A, et al. (2015) Breakdown of simple female genital fistula repair after 7 day versus 14 day postoperative bladder catheterisation: a randomised, controlled, open-label, non-inferiority trial. The Lancet 386: 56–62. doi: 10.1016/S0140-6736(14)62337-0 25911172

